# Can public health reconcile profits and pandemics? An analysis of attitudes to commercial sector engagement in health policy and research

**DOI:** 10.1371/journal.pone.0182612

**Published:** 2017-09-08

**Authors:** Jeff Collin, Sarah E. Hill, Mor Kandlik Eltanani, Evgeniya Plotnikova, Rob Ralston, Katherine E. Smith

**Affiliations:** 1 Global Public Health Unit, Social Policy, School of Social and Political Science, University of Edinburgh, Edinburgh, United Kingdom; 2 Information Services Division, NHS National Services Scotland, Gyle Square, 1 South Gyle Crescent, Edinburgh, United Kingdom; University of Glasgow, UNITED KINGDOM

## Abstract

**Background:**

Public health’s terms of engagement with unhealthy commodity industries (alcohol, tobacco and ultra-processed food and drinks) have become increasingly contested in policy and research. We sought to identify approaches that could attract consensus support within and across policy domains.

**Methods:**

Using snowball sampling, we undertook an online survey of 335 health researchers, advocates and policymakers, in 40 countries, assessing responses to stated principles, claims and recommendations for engaging with unhealthy commodity industries in relation to key policy and research initiatives.

**Results:**

Most respondents identified a fundamental conflict between industry interests and public health objectives for all three industries, with agreement greatest in relation to tobacco and weakest for food. This pattern was replicated across diverse questions regarding potential forms of engagement, including in rejecting voluntarism and partnership approaches to health policy. While awareness of tobacco industry tactics to influence policy and research was higher than for alcohol and food, most respondents rejected the view that the influence of the latter was less significant for public health. Proposals that health and research organisations should divest their funds attracted less support with respect to food, while restricting publication of industry-funded research in academic journals was the issue that most divided opinion. Respondents reported most difficulty in answering questions about the food industry.

**Conclusions:**

The strong consensus around restricting interactions with the tobacco industry supports increased implementation of the WHO Framework Convention on Tobacco Control’s conflict of interest provisions. There is strong support for the extension of such practices to the alcohol industry, challenging current norms. More mixed responses indicate a need for greater clarity in defining the food industry, and for research analyzing links, similarities and differences across different types of unhealthy commodity producers. Partnership approaches to addressing non-communicable diseases seem incapable of attracting widespread support across public health, challenging practice in many contexts.

## Background

Non-communicable disease (NCD) epidemics are increasingly recognised as being driven by transnational corporations involved in the production, manufacture and retail of unhealthy commodities. This suggests that the extensive institutional and financial links between such corporations and public health researchers, non-governmental organisations (NGOs), and health and development agencies are characterised by complex and potentially conflicting interests. [1] These complexities are highlighted via the increased prominence of NCDs in the global health agenda since the 2011 UN High Level Meeting; [2] for example, concerns about the terms on which public health interacts with industries such as alcohol and ultra-processed food and drinks have contributed to the tortuous progress of WHO’s proposed framework for engagement with non-state actors. [3,4] Such tensions are also evident in many national contexts, epitomised in England by the withdrawal of health advocates from government convened Public Health Responsibility Deals (PHRD) with private sector actors, including the alcohol and food industries. [5]

Underlying these debates is a distinctive pattern of practices and norms within tobacco control policy and research that, conversely, seeks to minimise industry engagement. 6] These practices mean that manufacturers of tobacco products are often explicitly excluded from both the making of health policy and the conduct of health research. This exclusion is embedded in the WHO Framework Convention on Tobacco Control (FCTC), which starkly contrasts with the partnership, voluntarism and self-regulatory approaches widely employed in strategies to reduce dietary and alcohol related ill-health. FCTC Article 5.3, for example, requires parties to protect health policymaking from tobacco industry interference. [7] Tobacco industry interactions with health researchers are widely rejected, given extensive evidence of industry manipulation of science, [8,9] and some major funders and medical publishers have adopted restrictions regarding industry sponsored research. [10,11]

A longstanding depiction of the fundamental conflict of interest between the tobacco industry and public health as unique—‘tobacco exceptionalism’ [6]—has been associated with reticence to extend such practices to the alcohol and food industries. Yet this exceptionalism is being challenged amid calls for framework conventions to address alcohol or nutrition [12–14] and other strategies to limit opportunities for industry engagement in policymaking. [1] Interest in exploring more cohesive policy approaches across these industries is further stimulated by emerging evidence of strategic similarities, [15–17] and by their comparable global health impacts [1,18] and economic and social costs. [1,19]

Research to date has not, however, explored support within public health for extending the practices and norms of tobacco control to other contexts, or for retaining divergent policy approaches. Following requests from health advocates to consider scope for a consensus statement on engagement between public health and unhealthy commodity industries, we sought to address this gap via a scoping survey of public health academics, advocates and policymakers. Here, we focus on three key aspects of the findings. First, we map the extent of variation within the public health community in perceptions of the alcohol, food, and tobacco industries. Second, we examine popular rationales for distinctions between the three. Finally, we consider whether the results indicate an emerging consensus around specific policies and practices for commercial sector engagement in, or exclusion from health policy and research.

## Methods

An online scoping survey was conducted to assess the views of health researchers, advocates and policymakers regarding appropriate forms of engagement between public health and unhealthy commodity industries. The survey’s introduction explained that we were focusing on attitudes to policy and research engagement with manufacturers of tobacco, alcohol and ultra-processed food and drink products [1] (abbreviated in the questions to ‘tobacco’, ‘alcohol’ and ‘food’: see S1 File—Survey Questions). Rather than reflecting the views of a clearly-defined group of respondents, this survey was intended as a scoping exercise within a loosely-categorised sample of self-identified public health professionals with a stated interest in prevention of NCDs. Based on the contact lists of the authors and an expert panel, and using a snowball approach to recruitment, the survey aimed to provide a broadly descriptive account of the range of views held by those participating, recognising that these respondents come from a broad range of geographical and professional contexts within public health.

The survey instrument was developed using the online software tool SurveyExpression. The survey questionnaire presented respondents with a series of key claims and recommendations regarding the relationship between public health and the three industry groups (see S1 File–Survey Questions). These claims were drawn from the academic literature, [1] key national and international policy initiatives, [20,21] and (with reference to approaches used in tobacco control) the survey drew on guidelines and policies to restrict tobacco industry interference in policy and research. [10–11,22]

A pilot version of this questionnaire was reviewed by 12 experts working on alcohol, food and tobacco-related health issues across academic and policy contexts in diverse countries. This group of individuals was identified via investigators’ professional networks, relevant academic, research and policy institutions. The panel’s comments were used to refine the questionnaire and develop an accompanying webpage [23] to provide further information for potential survey participants as well as explanatory comments on contested terms employed in survey questions. This research was approved by the School of Social & Political Science's Research Ethics Subcommittee at the University of Edinburgh on the basis that it met Level 1 requirements (i.e. that the research entailed no substantive ethical risks that could be reasonably foreseen).

The final version of the questionnaire included 23 individual questions (see S1 File). It explored responses across four areas: broad *principles* for public health’s engagement with the tobacco, alcohol and food industries; respondents’ awareness of *industry tactics to influence policy*; *recommendations* for appropriate engagement with these industries in health governance; and *cross-industry comparisons and distinctions* (focusing on understandings of similarities and differences with tobacco). Within each of these areas, questions were organised to allow comparison of responses across the three industry categories (tobacco, alcohol and food). Respondents could elect not to answer any particular question. A final page asked whether respondents had experienced particular difficulty in answering questions on any one industry. Respondents were also invited to provide information on their geographical and institutional location, and their professional role.

Our target ‘population’ for the survey was broadly conceived as members of the global public health community with a particular interest in prevention of NCDs–including scholars, advocates and policymakers whose work relates to health aspects of tobacco, alcohol and ultra-processed food and beverages. Since there is obviously no universal register of such diverse individuals, we necessarily employed a non-random sample to distribute the questionnaire, using existing contact lists from amongst the authors and the expert group and inviting respondents to forward the survey on to colleagues to whom it might be of interest. There was no intention to conduct a representative survey of experts across the world. Use of a non-random sampling frame was judged to be appropriate given the exploratory nature of the study, and the absence of a practical alternative. Given that this area is poorly researched, a mapping study which identifies existing perceptions (rather than attempting to evaluate their prevalence in the whole expert community) was considered as a pertinent initial step.

In July 2015 the survey was distributed via e-mail across professional network contacts, both in the UK and internationally, and via relevant groups such as the UK Centre on Tobacco and Alcohol Studies, Alcohol Health Alliance’s newsletter, and Canada’s Centre for Science in the Public Interest and the Politics of Health listserve. We opportunistically sought views from a wide range of public health scholars, advocates and policymakers working in diverse geographical contexts, recognising that no formal sampling frame exists for such diverse communities. Respondents were encouraged to distribute the survey as they saw fit, with no explicit restrictions. As response rates slowed, a final round of reminders was sent and the survey was closed in September 2015.

**Fig 1:**
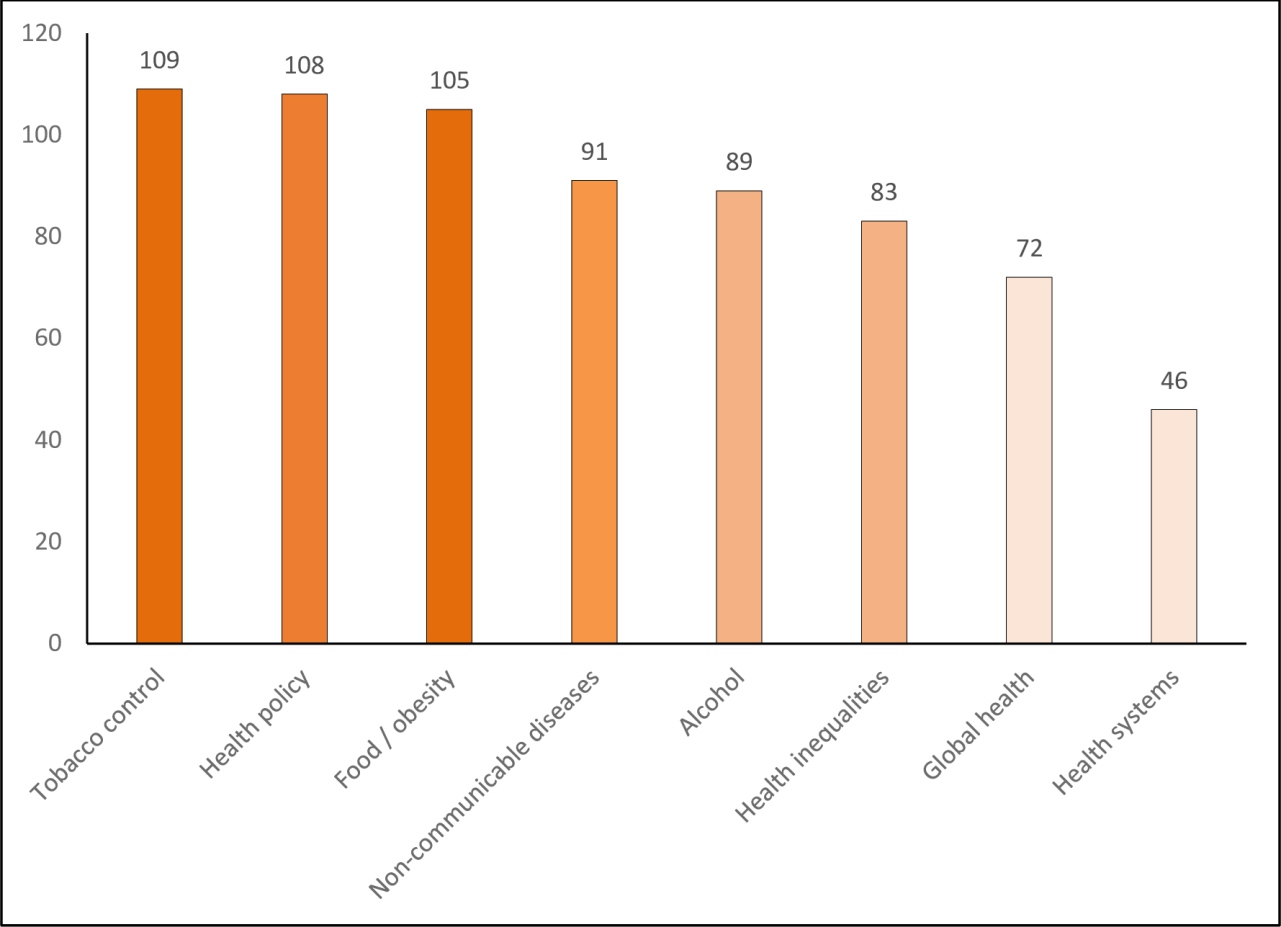
Foci of respondents current work (number of respondents in each category).

**Fig 2:**
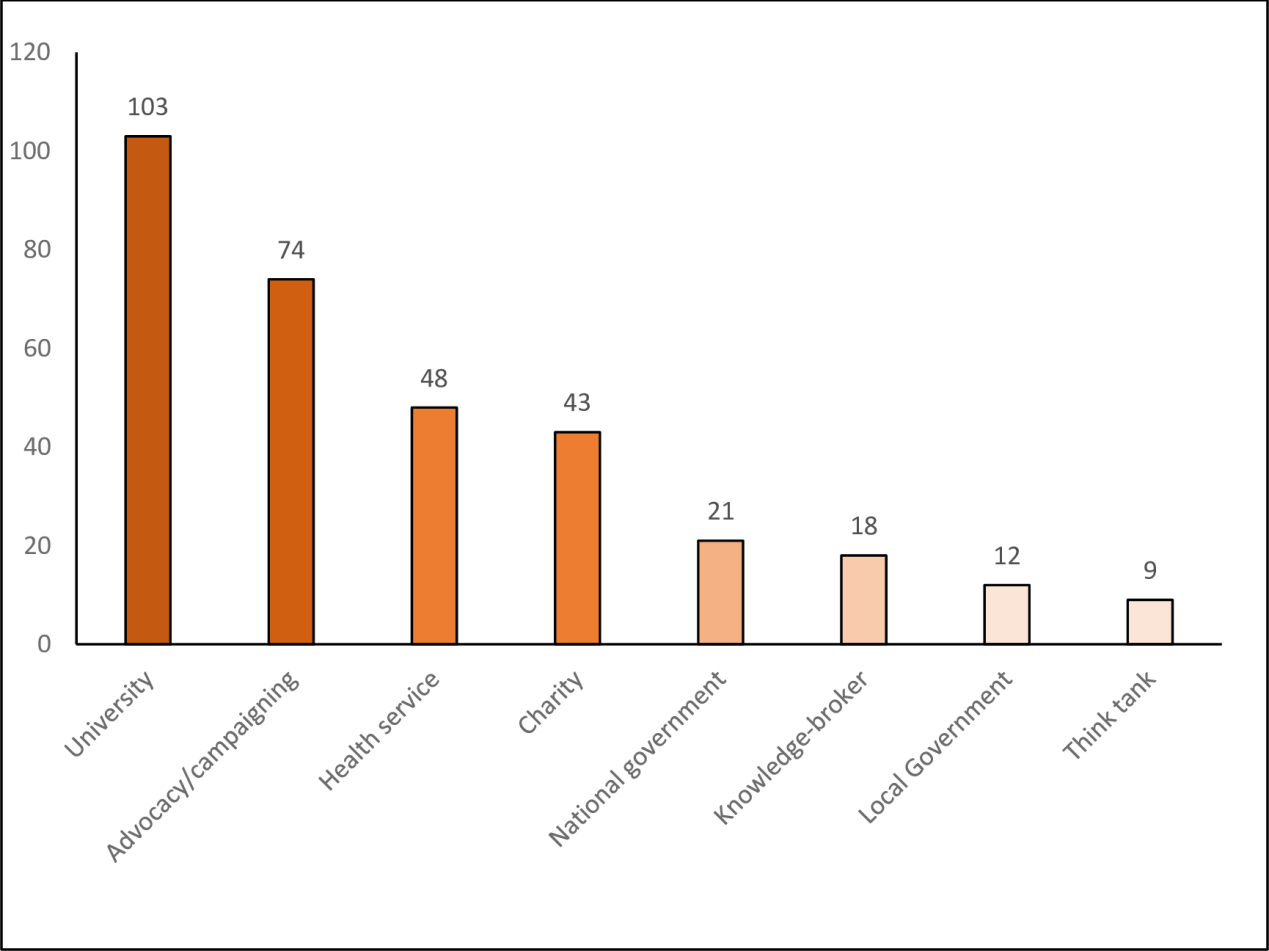
Institutional affiliation of respondents (number of respondents in each category).

A total of 335 respondents completed the survey, with specific item responses ranging from 245 to 330. Survey respondents worked in over 40 countries, with the UK and Canada being most frequently cited, followed by Australia and the USA, and with substantive contributions from respondents across Africa, Asia and Latin America (with at least 16 responses from each of those regions). The diverse foci of current work and institutional affiliations identified by respondents are indicated in Figs 1 and 2.

As with any non-probability sample, we cannot estimate the degree to which our results are generalizable to the wider population of researchers, advocates and policymakers. However, findings of this study can provide an insight into existing attitudes to commercial sector engagement in health policy and research and can be used to inform further research in this area, for instance for generating hypotheses and testing them on a wider expert population by means of random sampling designs.

## Results

As noted above, a total of 335 respondents completed the survey, with response rates for individual questions ranging from 73–99% (see S2 File–Results Frequency Distribution for the full aggregate survey results including numbers responding to each item).

### Principles of industry engagement in health policy

**Fig 3:**
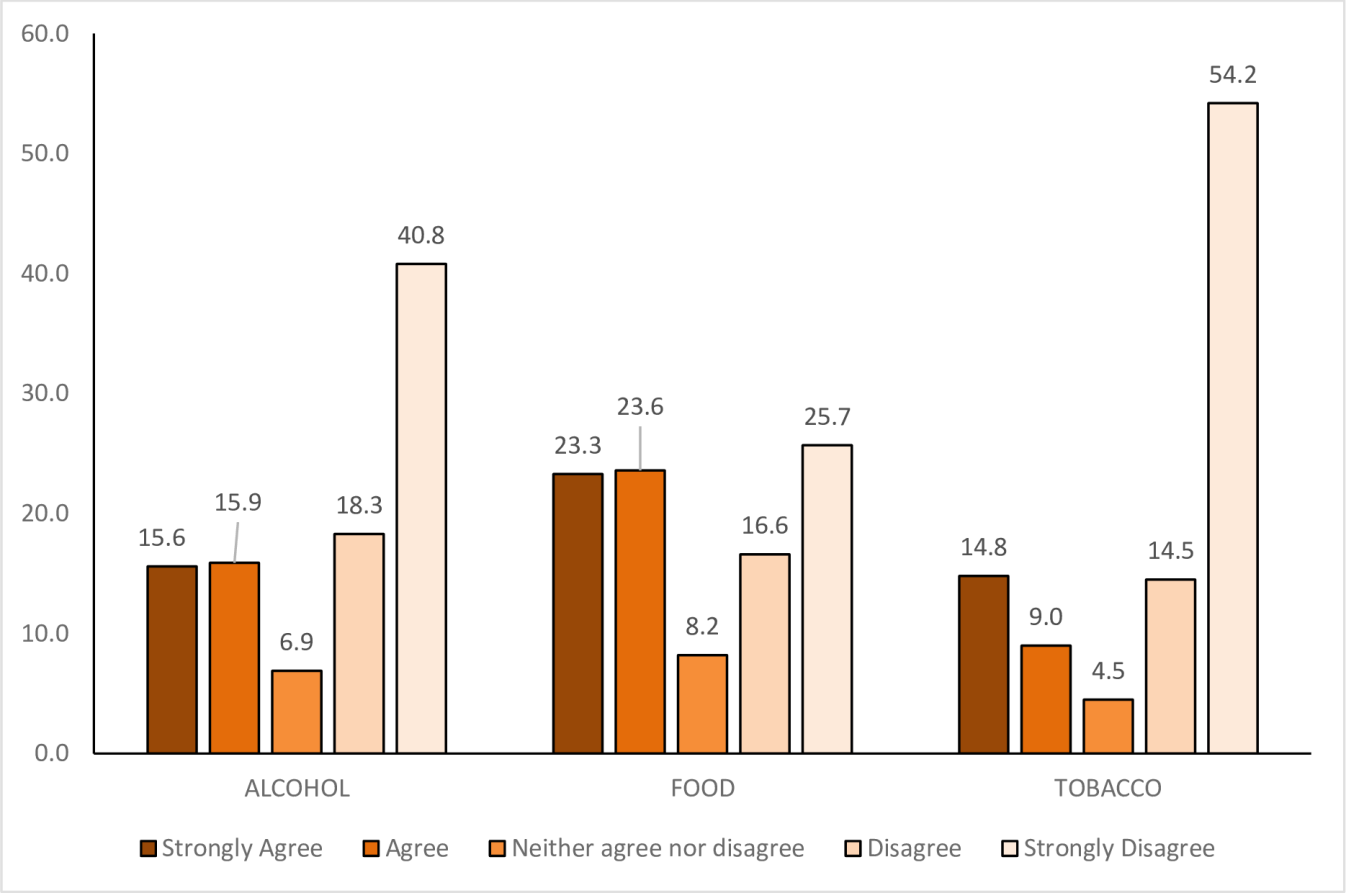
Level of agreement with the statement. “Enabling the active participation of all key stakeholders, including this industry, is vital to effective health governance” regarding Alcohol, Food, and Tobacco (percentage of respondents choosing each category).

Reflecting contemporary understandings of ‘good governance’, [24] an initial question sought responses to the claim that active participation of all key stakeholders, including industry, is vital to effective health governance. While a clear majority of respondents opposed the participation of tobacco and alcohol industries in health governance, there was a more mixed response in relation to the food industry (Fig 3).

A substantial majority of respondents identified a ‘fundamental and irreconcilable conflict between industry interests and public health objectives’ in relation to all three industries, although the proportion of participants agreeing with this statement varied from over 90% for tobacco to around two-thirds in the case of food. The argument that public health researchers, advocates and practitioners should not accept funding from the tobacco industry was correspondingly supported by around 90% (comparing with 86% and 72% for alcohol and food industries respectively). There was near unanimous support for the adoption of policies to improve accountability and transparency in interactions between public sector employees and these industries, with around 95% of respondents agreeing with this statement in each of the three contexts.

The dual claim that ‘tobacco is a special case, and the principle of precluding partnership and minimising engagement should not be extended to [alcohol / food]’ induced a mixed response, with respondents’ comments suggesting this partly reflected uncertainty in interpreting the claim. Nevertheless, a majority of respondents to this question disagreed with the statement in relation to both alcohol and food, suggesting broad interest in the potential application of practices developed in tobacco control to manage interactions with other unhealthy commodity industries.

### Industry tactics to influence policy

The survey sought to examine awareness of a range of industry tactics to shape policies, adapted from a WHO list of tactics employed by the tobacco industry. [25] Three broad patterns are evident in responses here (see Table 1). First, for routine activities that most industries might be expected to utilise (philanthropy, corporate social responsibility, public relations, employing consultants, lobbying) there was minimal variation in respondents’ awareness of behaviours across the three industries. Second, for activity descriptions that may imply more questionable or potentially deceptive use of financial resources to exert influence (direct funding of politicians or parties; creation or funding of alliances or front groups; funding research to create or maintain doubt about health implications; and ‘revolving door’ recruitment of key officials or politicians), respondents were slightly more likely to be aware of these tactics in relation to the tobacco industry than with alcohol or food. Third, awareness of the use of litigation, trade agreements and intimidation of opponents was much more strongly associated with the tobacco industry (with around twice as many respondents aware of evidence in relation to tobacco than to alcohol or food industries).

**Table 1:** Percentage of respondents indicating awareness of evidence for specific industry tactics to influence health policy (total N: 335).

	ALCOHOL	FOOD	TOBACCO
Employ consultants	50.6	45.8	53.0
Create or fund alliances or front groups	49.4	40.2	55.1
Direct political funding of parties and/or politicians	39.0	33.9	44.0
Funding of health promotion activities and/or pre-emption of policies	49.4	52.7	40.5
Funding research to create/maintain doubt about health implications	40.5	43.5	54.8
Intimidation of individuals or organisations that favour regulation of industry/product	23.8	17.0	42.3
Lobbying	58.3	56.3	59.8
Recruit key government officials or politicians (revolving door)	35.4	30.7	39.6
Philanthropy	45.5	44.9	42.6
Corporate social responsibility	48.5	47.3	44.3
Public relations / efforts to shape public perceptions of industry and/or its products	57.7	57.7	55.7
Litigation	28.0	21.1	50.6
Use of trade agreements	26.8	25.3	43.2

Interestingly, one tactic was more strongly associated with alcohol and food companies than the tobacco industry; only around one quarter of respondents were aware of funding of health promotion activities or pre-emption of policies by the tobacco industry, though both have long been documented. [25]

### Appropriate engagement in health governance

Opinions on how best to manage engagement with the three industries were sought across four broad types of recommendation. First, respondents were presented with positive assertions of the merits of voluntarism and partnership with the private sector. There was very limited support for the claim that voluntary agreements with industry would enable public health to progress more rapidly than via legislation (ranging from under 5% for tobacco to 14% for food). There was modest support for promoting multi-stakeholder partnerships with industry to allow access to its resources and expertise; 28% agreed with respect to the food industry, under 20% for alcohol and 13% for tobacco. Alongside some support for partnerships with the food industry, however, it is worth noting that strong disagreement with this proposal was still by far the most popular response.

Second, respondents were asked about proposals on restricting industry engagement in policy. Excluding industry from policy formulation but recognising roles in implementation [1] attracted majority support in all three cases. Substantial majorities also supported the suggestion that public and third sector organisations should reject partnerships and non-binding agreements with the tobacco, alcohol and food industries, with a near identical pattern of agreement that the public health community should not engage with social responsibility initiatives.

Third, a series of claims advocating abandonment of financial links, both in terms of investments and research funding, elicited a strong consensus across alcohol and tobacco that was less clear in relation to the food industry. Full divestment of funds by, firstly, health charities, public sector organisations and health campaigning organisations and, secondly, universities and other higher education institutions, was strongly supported by around two-thirds of respondents for the alcohol industry and over three-quarters for tobacco, but in both cases less than 40% strongly agreed in relation to food. A similar pattern characterised responses to suggestions that universities should refuse to accept funding from these industries and that health journals should refuse to publish industry-funded research; around two-thirds agreed in relation to alcohol, three quarters in relation to tobacco companies but just over half for the food industry.

Responses to a contrasting claim, that academic journals should publish industry-funded research subject to requirements regarding declaration of interests, suggests high levels of division and (given preceding responses) inconsistency. Around half agreed with academic journals publishing research funded by the food industry on this basis, while agreement also slightly outnumbered disagreement in relation to alcohol. This was the statement in the survey that most divided opinion in relation to the tobacco industry, with around 40% agreeing with publication of industry-funded research and around half disagreeing.

### Cross-industry comparisons

The final section of the survey examined the extent to which respondents regarded the influences of the alcohol and food industries on policy and research as less significant for public health than for tobacco. The food and alcohol industries were seen as less important than tobacco in this context by around one third and one quarter of respondents respectively, with small majorities disagreeing in both cases.

Those respondents identifying either the food or alcohol industry as being less significant to public health were then asked to select from a list of possible explanations. In relation to food, the three most popularly cited reasons were that food products were seen as less harmful at the individual level, less harmful at the population level and as more socially acceptable in the respondent’s work context, with each selected by more than half of these respondents. In relation to alcohol, explanations appear more diffuse. Only the perception that alcohol products are less harmful than tobacco at the individual level was selected by a majority of such respondents, followed by lower recognition of evidence of the alcohol industry seeking to mislead the public and policymakers, and the identification of alcohol products as less harmful at a population level.

### Comments on survey instrument

Finally, respondents were able to indicate whether they had experienced difficulties in answering questions relating to any of these industries. Over a third of respondents here reported no particular difficulty, while very few of those reporting difficulties cited the tobacco industry. In contrast, 14% found it difficult to answer questions on alcohol and over one quarter cited difficulties relating to food industry questions. When asked to describe such difficulties, those citing the alcohol industry referred primarily to limited knowledge of or engagement with alcohol issues, uncertainty over health harms, or to working in national contexts where the formal alcohol industry is restricted. Answers in relation to the food industry were more diverse and complex, with the most frequently cited reasons being food’s status as a necessity, alongside a series of closely related uncertainties over how best to define the food industry, perceptions that its structure is more diverse than those of alcohol or food, and that it comprises beneficial products and actors as well as harmful ones.

### Study limitations

We necessarily employed a non-random sampling strategy in distributing the survey questionnaire. Since there is no universal register of our target population (members of the global public health community with a particular interest in prevention of NCDs), we were reliant on existing contact lists and snowball sampling. Undoubtedly, we were not able to contact all members of our target population: some eligible individuals may have received multiple invitations to participate in the survey, while many others would not have been contacted at all. Similarly, we have no way of estimating what proportion of eligible respondents were included in the survey, or what proportion of those invited actually participated. We are therefore unable to calculate some parameters normally associated with survey-based research, such as the survey response rate, and the results of this pilot have limited generalisability.

Nevertheless, we are confident our sample provides non-proportionate representation of key groups, including public health professionals working in a range of roles and geographical contexts. While our results cannot be generalised to the whole public health community, we believe the descriptive account of the results from this scoping survey provides valuable information about the range of views that exist among those with an interest in NCD prevention and a qualitative sense of the extent to which these view diverge or converge in relation to key issues.

We are unable to locate any previous research exploring the views of diverse public health professionals regarding appropriate terms for engagement with the producers of unhealthy commodities (beyond tobacco), nor assessing the extent to which there may be enthusiasm or reluctance for extending the practices and norms of tobacco control to other contexts. Given the scarcity of evidence in this area, we believe this scoping survey makes a valuable contribution by mapping the range of existing perceptions within public health and providing a broad sense of the extent–or absence–of consensus around specific principles and approaches.

## Discussion and conclusions

This survey examined attitudes within the public health community to managing the terms of engagement with alcohol, food and tobacco industries in health policy and research. Perhaps its most predictable aspect is the very strong consensus around measures to pro-actively restrict the terms of any interaction with the tobacco industry. The consistency with which the language of multi-stakeholder partnership is rejected, and the near unanimity with which health objectives and tobacco industry interests are seen as incompatible, demonstrates strong support for the principles of protecting health policy from tobacco industry interference. This suggests that efforts to improve on the rather limited track record of many governments in implementing FCTC Article 5.3 [26] are likely to be well supported within public health.

There are, nonetheless, two interesting caveats. First, the majority of respondents agreed that while the tobacco industry should be excluded from policy formulation, it could have a significant role in policy implementation. While the terms of this ‘significant role’ weren’t specified or explored, it may suggest willingness for more expansive engagement than that recommended in Article 5.3 implementation guidelines’ call to “protect the formulation *and implementation* of public health policies for tobacco control from the tobacco industry to the greatest extent possible” (emphasis added). [22] Secondly, the survey suggests a level of ongoing uncertainty or disquiet regarding policies around publication of research funded by the tobacco industry.

Viewed from an alcohol policy perspective, the survey responses provide a consistent pattern by which approaches to restrict engagement with the alcohol industry are supported almost as widely as for the tobacco industry. The widely shared perceptions of the alcohol and tobacco industries, and common attitudes to minimising interactions depicted above, contrasts with stark divergence in regulatory practice at national and international levels. If the survey offers encouragement to those making the case for expansion of tobacco control policies and practices to alcohol, it also highlights the need to increase awareness of industry tactics to influence policy and of alcohol-related harm at a population level. In the context of recent European legal challenges by alcohol industry actors to the implementation of minimum unit pricing for alcohol in Scotland, [27] health advocates might have hoped that more than a quarter of respondents would identify litigation as a tactic used by this industry.

Survey findings offer the least clarity on how to manage interactions between public health and the ‘food industry’. While the introduction to our survey specified that in this context we were referring to “the parts of this sector that manufacture ultra-processed food and drinks”, this ambiguity highlights widespread uncertainty about how to define this industry and/or how to differentiate between those of its actors viewed as capable of contributing positively to population health and those that are not. In relation to the food industry, statements about regulatory strategies consistently follow similar patterns to those for tobacco and alcohol but exhibit lower levels of consensus. This is particularly so with respect to funding and research, where there were substantial gaps between proportions advocating divestment of tobacco or alcohol shares compared with the food industry, and much more limited support for non-publication of research funded by the food industry.

The above difficulties and differences should not, however, disguise the extent to which perspectives and preferences are held in common across all three industries, nor the scale of respondents’ divergence from politically dominant commitments to partnership. While sympathy for engagement is greater with respect to the food industry, a very positively worded statement on the importance of active participation of all key stakeholders to effective health governance did not receive majority endorsement for any industry. By contrast, a majority of respondents supported statements identifying food industry interests as being fundamentally in conflict with public health, rejecting partnerships and non-binding agreements, and advocating the industry’s exclusion from policy formulation.

All of this demonstrates the lack of support within public health for policy approaches involving partnership working and voluntary agreements, such as England’s Public Health Responsibility Deals and WHO’s Global Coordination Mechanism for NCDs. Given the importance of clear policy objectives to effective health advocacy, [28–29] these indications of consensus within the public health community suggest that core elements of national and international strategies to reduce NCDs are likely to remain contentious.

To tackle this impasse, we urgently need a research agenda capable of a more nuanced analyses of unhealthy commodity producers and their engagement in health policy and research. Such an agenda needs to more carefully define industries, particularly for food, and consider the case for delineating particular kinds of actors within industries (e.g. those operating on a small-scale where public health impacts may be negligible, or larger actors whose overall product portfolio might be considered neutral or positive in health terms). Research is also needed to examine structural links between companies and across industries, epitomised, for example, by the brewing giant SAB Miller being part-owned by Altria (producers of Marlboro cigarettes) and having a major distribution deal with Coca Cola. [30] Such interpenetration calls into question governance practices that distinguish between tobacco and other unhealthy commodity industries, pre-supposing that these can be clearly differentiated.

Importantly, there is an apparent consensus within public health that the interests of alcohol and food manufacturers fundamentally conflict with public health objectives. Given this widespread recognition, it seems clear that health governance and research require more coherent approaches to the terms with which they engage with unhealthy commodity producers. This could involve examining how the adaptation of tobacco control practices and norms, including those arising from Article 5.3, [22] might inform measures to improve transparency and governance across NCD policy debates.

## Supporting information

S1 File Survey questions. (PDF)

S2 File Results frequency distributions. (PDF)
