# Supplementary material for: Can public health reconcile profits and pandemics? An analysis of attitudes to commercial sector engagement in health policy and research
Source: PLoS One. 2017 Sep 8;12(9):e0182612. doi: 10.1371/journal.pone.0182612 (PMC5590731; doi:10.1371/journal.pone.0182612)
Supplement: S1 File — (PDF) [file pone.0182612.s001.pdf]

S1 File. Survey Questions

Commercial Sector Engagement in Health Policy and Research

1. Introduction
This survey is being conducted by researchers in the Global Public Health Unit, University of Edinburgh. It is designed to explore where there is consensus within the public health community around policies and practices relating to commercial sector engagement with research and policy. The survey focuses on the alcohol industry, the food industry (by which we mean the parts of this sector that manufacture ultra-processed food and drinks) and the tobacco industry. This focus reflects the clear health impacts of their respective products on premature morbidity and mortality, and for each industry our attention centres on leading MANUFACTURERS of these products (rather than encompassing a more diverse range of actors such as retailers and distributors).

It should take not more that 10-15 minutes to complete this survey. All questionnaire responses are anonymous.

For more information about this survey please go to: http://tinyurl.com/n9o2ujj

2. Principles
With reference to the ALCOHOL, FOOD and TOBACCO industries, to what extent do you agree with each of the following claims?

1. Enabling the active participation of all key stakeholders, including this industry, is vital to effective health governance.

	Strongly Agree	Agree	Neither agree nor disagree	Disagree	Strongly Disagree	Don't know
ALCOHOL	○	○	○	○	○	○
FOOD	○	○	○	○	○	○
TOBACCO	○	○	○	○	○	○

2. Policies should be adopted to improve accountability and transparency in any interaction between public sector employees and this industry on health policy and research.

	Strongly Agree	Agree	Neither agree nor disagree	Disagree	Strongly Disagree	Don't know
ALCOHOL	○	○	○	○	○	○
FOOD	○	○	○	○	○	○
TOBACCO	○	○	○	○	○	○

3. There is a fundamental and irreconcilable conflict between industry interests and public health objectives.

	Strongly Agree	Agree	Neither agree nor disagree	Disagree	Strongly Disagree	Don't know
ALCOHOL						
FOOD						
TOBACCO						

4. This industry should not receive preferential treatment, such as tax breaks and other financial incentives, that may conflict with public health goals.

	Strongly Agree	Agree	Neither agree nor disagree	Disagree	Strongly Disagree	Don't know
ALCOHOL						
FOOD						
TOBACCO						

5. Government officials should not promote the interests of this industry overseas (e.g. by lobbying foreign governments on their behalf).

	Strongly Agree	Agree	Neither agree nor disagree	Disagree	Strongly Disagree	Don't know
ALCOHOL						
FOOD						
TOBACCO						

6. Public health researchers, advocates and practitioners should not accept funding from this industry.

	Strongly Agree	Agree	Neither agree nor disagree	Disagree	Strongly Disagree	Don't know
ALCOHOL						
FOOD						
TOBACCO						

7. **Tobacco is a special case, and the principle of precluding partnership and minimising engagement should not be extended to this industry**.

	Strongly Agree	Agree	Neither agree nor disagree	Disagree	Strongly Disagree	Don't know
ALCOHOL	○	○	○	○	○	○
FOOD	○	○	○	○	○	○

8. **Any comments on this section? (entirely optional)**.

3. Industry tactics to influence policy

1. Do you know of any evidence that the ALCOHOL, FOOD and TOBACCO industries has employed any of these tactics to achieve favourable policy outcomes?

**Where you are aware of such evidence, please tick the relevant box. Please leave all other boxes blank.*

	ALCOHOL	FOOD	TOBACCO
Employ consultants	☐	☐	☐
Create or fund alliances or front groups	☐	☐	☐
Direct political funding of parties and/or politicians	☐	☐	☐
Funding of health promotion activities and/or pre-emption of policies	☐	☐	☐
Funding research to create/maintain doubt about health implications	☐	☐	☐
Intimidation of individuals or organisations that favour regulation of industry/product	☐	☐	☐
Lobbying	☐	☐	☐
Recruit key government officials or	☐	☐	☐

politicians (revolving door)			
Philanthropy	☐	☐	☐
Corporate social responsibility	☐	☐	☐
Public relations / efforts to shape public perceptions of industry and/or its products	☐	☐	☐
Litigation	☐	☐	☐
Use of trade agreements	☐	☐	☐

2. **Any comments on this section? (entirely optional).**

4. Recommendations

To what extent do you agree that the following recommendations apply to the specified industries:

*If you are not able to respond to any of these questions, please leave them blank.

1. **Voluntary agreements with this industry will enable public health to progress more rapidly than statutory approaches.**

	Strongly Agree	Agree	Neither agree nor disagree	Disagree	Strongly Disagree	Don't know
ALCOHOL	○	○	○	○	○	○
FOOD	○	○	○	○	○	○
TOBACCO	○	○	○	○	○	○

2. **Multi-stakeholder partnerships involving this industry should be promoted to enable access to its knowledge, expertise, technology and financial resources.**

	Strongly Agree	Agree	Neither agree nor disagree	Disagree	Strongly Disagree	Don't know
ALCOHOL	○	○	○	○	○	○
FOOD	○	○	○	○	○	○
TOBACCO	○	○	○	○	○	○

3. **This industry should be excluded from the formulation of public health policies, but can have a significant role in policy implementation.**

	Strongly Agree	Agree	Neither agree nor disagree	Disagree	Strongly Disagree	Don't know
ALCOHOL						
FOOD						
TOBACCO						

4. **Public and third sector organisations should reject partnerships and non-binding agreements with this industry.**

	Strongly Agree	Agree	Neither agree nor disagree	Disagree	Strongly Disagree	Don't know
ALCOHOL						
FOOD						
TOBACCO						

5. **The public health community should not engage with this industry's social responsibility initiatives.**

	Strongly Agree	Agree	Neither agree nor disagree	Disagree	Strongly Disagree	Don't know
ALCOHOL						
FOOD						
TOBACCO						

6. **Health charities, public sector organisations and health campaigning organisations should fully divest their funds from this industry.**

	Strongly Agree	Agree	Neither agree nor disagree	Disagree	Strongly Disagree	Don't know
ALCOHOL						
FOOD						
TOBACCO						

7. **Universities and other higher education institutions should fully divest their funds from this industry.**

	Strongly Agree	Agree	Neither agree nor disagree	Disagree	Strongly Disagree	Don't know
ALCOHOL						
FOOD						
TOBACCO						

8. **Universities and other higher education institutions should refuse to accept funding from this industry.**

	Strongly Agree	Agree	Neither agree nor disagree	Disagree	Strongly Disagree	Don't know
ALCOHOL						
FOOD						
TOBACCO						

9. **Health journals should refuse to publish research funded by this industry.**

	Strongly Agree	Agree	Neither agree nor disagree	Disagree	Strongly Disagree	Don't know
ALCOHOL						
FOOD						
TOBACCO						

10. **Academic journals should publish research funded by this industry, subject to standard requirements regarding declaration of interests.**

	Strongly Agree	Agree	Neither agree nor disagree	Disagree	Strongly Disagree	Don't know
ALCOHOL						
FOOD						
TOBACCO						

11. **Any comments on this section? (entirely optional)**.

5. Cross-industry comparisons and distinctions

1. **Do you agree with the following claim: *'The influence of the ALCOHOL industry on policy and research is less significant for public health than that of tobacco'?***

- Yes (Go to question 2)
- No (Skip question 2, go to question 3)
- Do not know (Skip question 2, go to question 3)

2. I believe the influence of the ALCOHOL industry on policy and research is less significant for public health than that of tobacco because (select all those that apply):

- Alcohol products are less harmful than tobacco products at the individual level
- Alcohol products are less harmful than tobacco products at the population level
- Alcohol products are more socially acceptable in the contexts in which I work
- Alcohol products are less socially acceptable in the contexts in which I work
- I personally find alcohol products more acceptable/appealing
- There is less evidence that the alcohol industry has attempted to influence policy in ways which are damaging to health
- There is less evidence that the alcohol industry has attempted to influence research in ways which are damaging to health
- There is less evidence that the alcohol industry has sought to mislead the public / policymakers about the negative health and social consequences of its products.

3. **Do you agree with the following claim: *'The influence of the FOOD industry on policy and research is less significant for public health than that of tobacco'?***

- Yes (Go to question 4)
- No (Skip question 4, go to question 5)
- Do not know (Skip question 4, go to question 5)

4. **I believe the influence of the FOOD industry on policy and research is less significant for public health than that of tobacco because (select all those that apply):**

- Food products are less harmful than tobacco products at the individual level
- Food products are less harmful than tobacco products at the population level
- Food products are more socially acceptable in the contexts in which I work

- [ ] Food products are less socially acceptable in the contexts in which I work
- [ ] I personally find food products more acceptable/appealing
- [ ] There is less evidence that the food industry has attempted to influence policy in ways which are damaging to health
- [ ] There is less evidence that the food industry has attempted to influence research in ways which are damaging to health
- [ ] There is less evidence that the food industry has sought to mislead the public / policymakers about the negative health and social consequences of its products.

5. **Any comments on this section? (entirely optional).**

6. Personal experiences and views

1. **Institutional affiliation**

- [ ] University
- [ ] Knowledge-broker
- [ ] Charity
- [ ] Advocacy/campaigning
- [ ] Think tank
- [ ] Health service
- [ ] National government
- [ ] Local Government
- [ ] Other (Please Specify)

2. **Which of these would you describe as a focus of your public health work? (please tick all that apply):**

- [ ] Alcohol
- [ ] Food / obesity
- [ ] Global health

- [ ] Health inequalities
- [ ] Health systems
- [ ] Health policy
- [ ] Non-communicable diseases
- [ ] Tobacco control
- [ ] Other (Please Specify)

3. **In what country do you primarily work?**

4. **Are you engaged in research?**

- ○ Yes (Go to question 6)
- ○ No (Skip question 6, go to question 7)

5. If you are engaged in research, what methodological approach do you primarily employ? (please tick one):

- [ ] Qualitative methods
- [ ] Mixed methods
- [ ] Quantitative, non-experimental methods
- [ ] Quantitative, experimental methods
- [ ] Other (Please Specify)

6. **In political matters, people talk of "the left" and "the right." How would you place your views on this scale, generally speaking?**

○'1 Left'	○ '2'	○ '3'	○ '4'	○ '5'	○ '6'	○ '7'	○ '8'	○ '9'	○'10 Right'	○ Prefer not to say

7. **Are you affiliated with any of the following organisations/coalitions (tick all that apply)?**

- [ ] Conflict of Interest Coalition
- [ ] Framework Convention Alliance
- [ ] Global Alcohol Policy Alliance (GAPA)

- [ ] NCD Alliance
- [ ] UK Centre for Tobacco and Alcohol Studies (UKCTAS)
- [ ] World Health Organization

8. **Have you ever worked in any of the following industries? (tick all that apply)**

- [ ] Alcohol
- [ ] E-cigarettes
- [ ] Food and beverage
- [ ] Pharmaceutical
- [ ] Tobacco

9. **Do you have any formal relationship with, or financial investments in, any of the following industries? (tick all that apply)**

- [ ] Alcohol
- [ ] E-cigarettes
- [ ] Food and beverage
- [ ] Pharmaceutical
- [ ] Tobacco

10. **In the past ten years, have you received funding from any of the following sources for any research or other work relating to public health that you have undertaken / been professionally involved with (tick all that apply)?**

- [ ] Alcohol industry
- [ ] E-cigarette industry
- [ ] Food and non-alcoholic beverage industry
- [ ] Publicly funded health services
- [ ] Local Government
- [ ] Health/medical charity/campaigning organisation
- [ ] Other charity/campaigning organisation
- [ ] Pharmaceutical industry
- [ ] Charitable foundation (such as Wellcome Trust, Bill & Melinda Gates Foundation
- [ ] Retail industry
- [ ] Tobacco industry

- [ ] National government
- [ ] Government funded research councils
- [ ] European Union
- [ ] International organisations
- [ ] Prefer not to say
- [ ] Other (Please Specify)

11. **Any comments on this section? (entirely optional).**

7. FINAL QUESTIONS

1. Have you found it particularly difficult to answer our questions in relation to any of these industries? (please tick all that apply).

- [ ] NO
- [ ] YES, FOR ALCOHOL
- [ ] YES, FOR FOOD
- [ ] YES, FOR TOBACCO
- [ ] YES, FOR E-CIGARETTES

2. If 'YES', can you briefly explain why?

3. Do you have any final comments on this survey overall?
(entirely optional).

8. THANK YOU!

We really appreciate you taking the time to complete this survey and would be happy to keep you updated regarding the results of this research and/or a follow up interview. If you would like us to do so, please tick an appropriate box and write your name and e-mail address below. Please note that this information will be disconnected from your other answers (i.e. providing these details does not compromise the anonymity of the information you have provided).
