# Supplementary material for: Can public health reconcile profits and pandemics? An analysis of attitudes to commercial sector engagement in health policy and research
Source: PLoS One. 2017 Sep 8;12(9):e0182612. doi: 10.1371/journal.pone.0182612 (PMC5590731; doi:10.1371/journal.pone.0182612)
Supplement: S2 File — (PDF) [file pone.0182612.s002.pdf]

S2 File. Results Frequency Distributions

1. INTRODUCTION

2. PRINCIPLES

1. Enabling the active participation of all key stakeholders, including this industry, is vital to effective health governance.

	Strongly Agree	Agree	Neither agree nor disagree	Disagree	Strongly Disagree	Don't know	Number of Respondents
ALCOHOL	15.6%	15.9%	6.9%	18.3%	40.8%	2.4%	333
FOOD	23.3%	23.6%	8.2%	16.6%	25.7%	2.7%	331
TOBACCO	14.8%	9.0%	4.5%	14.5%	54.2%	3.0%	332

2. Policies should be adopted to improve accountability and transparency in any interaction between public sector employees and this industry on health policy and research.

	Strongly Agree	Agree	Neither agree nor disagree	Disagree	Strongly Disagree	Don't know	Number of Respondents
ALCOHOL	82.6%	14.4%	0.6%	0.6%	0.6%	1.2%	334
FOOD	80.5%	16.8%	0.3%	0.0%	0.6%	1.8%	333
TOBACCO	82.9%	13.1%	0.6%	0.6%	0.9%	1.8%	328

3. There is a fundamental and irreconcilable conflict between industry interests and public health objectives.

	Strongly Agree	Agree	Neither agree nor disagree	Disagree	Strongly Disagree	Don't know	Number of Respondents
ALCOHOL	57.8%	26.8%	6.3%	5.7%	2.7%	0.6%	332
FOOD	36.9%	29.9%	15.1%	11.8%	5.4%	0.9%	331
TOBACCO	79.8%	13.3%	2.7%	1.5%	1.8%	0.9%	332

4. This industry should not receive preferential treatment, such as tax breaks and other financial incentives, that may conflict with public health goals.

	Strongly Agree	Agree	Neither agree nor disagree	Disagree	Strongly Disagree	Don't know	Number of Respondents
ALCOHOL	84.6%	9.0%	3.6%	1.8%	0.3%	0.6%	332
FOOD	69.6%	13.1%	8.2%	5.2%	2.4%	1.5%	329
TOBACCO	88.1%	7.6%	2.1%	0.9%	0.3%	0.9%	327

5. Government officials should not promote the interests of this industry overseas (e.g. by lobbying foreign governments on their behalf).

	Strongly Agree	Agree	Neither agree nor disagree	Disagree	Strongly Disagree	Don't know	Number of Respondents
ALCOHOL	68.3%	13.3%	8.2%	7.6%	1.2%	1.5%	331
FOOD	48.9%	14.5%	14.5%	13.6%	5.4%	3.0%	331
TOBACCO	82.5%	9.5%	4.6%	1.8%	0.3%	1.2%	325

6. Public health researchers, advocates and practitioners should not accept funding from this industry.

	Strongly Agree	Agree	Neither agree nor disagree	Disagree	Strongly Disagree	Don't know	Number of Respondents
ALCOHOL	69.0%	17.0%	5.8%	4.6%	3.0%	0.6%	329

FOOD	51.5%	20.9%	12.3%	8.6%	5.5%	1.2%	326
TOBACCO	78.3%	11.3%	3.7%	2.8%	3.1%	0.9%	327

7. Tobacco is a special case, and the principle of precluding partnership and minimising engagement should not be extended to this industry.

	Strongly Agree	Agree	Neither agree nor disagree	Disagree	Strongly Disagree	Don't know	Number of Respondents
ALCOHOL	18.8%	13.5%	8.5%	14.1%	35.1%	10.0%	319
FOOD	15.1%	15.1%	15.8%	18.3%	25.2%	10.4%	317

3. INDUSTRY TACTICS TO INFLUENCE POLICY

1. Do you know of any evidence that the ALCOHOL, FOOD and TOBACCO industries has employed any of these tactics to achieve favourable policy outcomes?

**Where you are aware of such evidence, please tick the relevant box. Please leave all other boxes blank.*

	ALCOHOL	FOOD	TOBACCO
Employ consultants	64.0%	58.1%	67.4%
Create or fund alliances or front groups	62.5%	51.3%	69.7%
Direct political funding of parties and/or politicians	49.1%	43.4%	55.8%
Funding of health promotion activities and/or pre-emption of policies	62.5%	67.0%	50.9%
Funding research to create/maintain doubt about health implications	51.3%	54.7%	69.3%
Intimidation of individuals or organisations that favour regulation of industry/product	30.0%	21.7%	53.6%
Lobbying	74.2%	71.5%	75.7%
Recruit key government officials or politicians (revolving door)	44.9%	39.0%	50.2%
Philanthropy	57.3%	57.3%	53.6%
Corporate social responsibility	61.4%	59.9%	56.2%
Public relations / efforts to shape public perceptions of industry and/or its products	73.0%	73.0%	70.4%
Litigation	35.6%	27.3%	64.4%
Use of trade agreements	34.1%	32.2%	55.1%
Number of Respondents			267

4. RECOMMENDATIONS

1. Voluntary agreements with this industry will enable public health to progress more rapidly than statutory approaches.

	Strongly Agree	Agree	Neither agree nor disagree	Disagree	Strongly Disagree	Don't know	Number of Respondents
ALCOHOL	1.9%	4.9%	8.6%	21.6%	59.3%	3.7%	268
FOOD	4.1%	9.4%	11.3%	19.9%	48.9%	6.4%	266
TOBACCO	2.6%	1.9%	5.2%	13.4%	73.2%	3.7%	269

2. Multi-stakeholder partnerships involving this industry should be promoted to enable access to its knowledge, expertise, technology and financial resources.

	Strongly Agree	Agree	Neither agree nor disagree	Disagree	Strongly Disagree	Don't know	Number of Respondents
ALCOHOL	4.5%	12.9%	9.8%	20.1%	45.8%	6.8%	264
FOOD	9.4%	18.8%	13.9%	17.7%	31.6%	8.6%	266
TOBACCO	3.8%	9.1%	4.9%	12.9%	62.4%	6.8%	263

3. This industry should be excluded from the formulation of public health policies, but can have a significant role in policy implementation.

	Strongly Agree	Agree	Neither agree nor disagree	Disagree	Strongly Disagree	Don't know	Number of Respondents
ALCOHOL	30.6%	26.8%	10.9%	13.6%	14.3%	3.8%	265
FOOD	24.7%	34.6%	10.6%	12.2%	11.8%	6.1%	263
TOBACCO	33.6%	19.5%	7.6%	11.1%	24.0%	4.2%	262

4. Public and third sector organisations should reject partnerships and non-binding agreements with this industry.

	Strongly Agree	Agree	Neither agree nor disagree	Disagree	Strongly Disagree	Don't know	Number of Respondents
ALCOHOL	60.1%	20.9%	7.5%	6.0%	2.2%	3.4%	268
FOOD	41.5%	20.8%	16.2%	10.9%	4.9%	5.7%	265
TOBACCO	72.5%	13.8%	5.2%	4.5%	1.5%	2.6%	269

5. The public health community should not engage with this industry's social responsibility initiatives.

	Strongly Agree	Agree	Neither agree nor disagree	Disagree	Strongly Disagree	Don't know	Number of Respondents
ALCOHOL	57.6%	22.5%	6.3%	8.9%	3.7%	1.1%	271
FOOD	40.4%	20.7%	16.7%	13.0%	5.6%	3.7%	270
TOBACCO	69.1%	17.6%	3.3%	4.4%	4.0%	1.5%	272

6. Health charities, public sector organisations and health campaigning organisations should fully divest their funds from this industry.

	Strongly Agree	Agree	Neither agree nor disagree	Disagree	Strongly Disagree	Don't know	Number of Respondents
ALCOHOL	62.9%	17.2%	9.0%	4.5%	3.0%	3.4%	267
FOOD	36.3%	17.2%	23.6%	11.2%	5.2%	6.4%	267
TOBACCO	76.8%	12.5%	4.8%	1.1%	2.6%	2.2%	271

7. Universities and other higher education institutions should fully divest their funds from this industry.

	Strongly Agree	Agree	Neither agree nor disagree	Disagree	Strongly Disagree	Don't know	Number of Respondents
ALCOHOL	63.4%	15.5%	10.2%	4.2%	3.8%	3.0%	265
FOOD	36.7%	15.4%	23.9%	11.2%	7.3%	5.4%	259
TOBACCO	77.1%	11.3%	5.6%	1.5%	2.3%	2.3%	266

8. Universities and other higher education institutions should refuse to accept funding from this industry.

	Strongly Agree	Agree	Neither agree nor disagree	Disagree	Strongly Disagree	Don't know	Number of Respondents
ALCOHOL	66.3%	14.7%	9.2%	6.2%	2.2%	1.5%	273

FOOD	39.6%	14.8%	22.6%	12.6%	5.9%	4.4%	270
TOBACCO	79.5%	9.2%	5.1%	3.3%	1.8%	1.1%	273

9. Health journals should refuse to publish research funded by this industry.

	Strongly Agree	Agree	Neither agree nor disagree	Disagree	Strongly Disagree	Don't know	Number of Respondents
ALCOHOL	44.6%	19.9%	12.7%	12.0%	6.9%	4.0%	276
FOOD	31.1%	16.1%	23.1%	15.0%	8.4%	6.2%	273
TOBACCO	56.4%	14.5%	8.7%	10.2%	6.5%	3.6%	275

10. Academic journals should publish research funded by this industry, subject to standard requirements regarding declaration of interests.

	Strongly Agree	Agree	Neither agree nor disagree	Disagree	Strongly Disagree	Don't know	Number of Respondents
ALCOHOL	17.5%	27.7%	9.1%	19.7%	21.9%	4.0%	274
FOOD	17.9%	31.9%	15.8%	15.8%	13.9%	4.8%	273
TOBACCO	17.2%	23.4%	7.0%	15.8%	33.3%	3.3%	273

5. CROSS-INDUSTRY COMPARISONS AND DISTINCTIONS

1. Do you agree with the following claim: *'The influence of the ALCOHOL industry on policy and research is less significant for public health than that of tobacco'?*	N of Respondents	% of Respondents
Yes (Go to question 2)	76	27.1%
No (Skip question 2, go to question 3)	162	57.9%
Do not know (Skip question 2, go to question 3)	42	15.0%
Number of Respondents	280	100.0%

2. I believe the influence of the ALCOHOL industry on policy and research is less significant for public health than that of tobacco because (select all those that apply):	N of Responses	% of Respondents
Alcohol products are less harmful than tobacco products at the individual level	45	52.9%
Alcohol products are less harmful than tobacco products at the population level	37	43.5%
Alcohol products are more socially acceptable in the contexts in which I work	34	40.0%
Alcohol products are less socially acceptable in the contexts in which I work	5	5.9%
I personally find alcohol products more acceptable/appealing	25	29.4%
There is less evidence that the alcohol industry has attempted to influence policy in ways which are damaging to health	30	35.3%
There is less evidence that the alcohol industry has attempted to influence research in ways which are damaging to health	26	30.6%
There is less evidence that the alcohol industry has sought to mislead the public / policymakers about the negative health and social consequences of its products.	42	49.4%
Number of respondents		85

3. Do you agree with the following claim: *'The influence of the FOOD industry on policy and research is less significant for public health than that of tobacco'?*	N of Respondents	% of Respondents
Yes (Go to question 4)	91	33.0%

No (Skip question 4, go to question 5)	140	50.7%
Do not know (Skip question 4, go to question 5)	45	16.3%
Number of Respondents	276	100.0%

4. I believe the influence of the FOOD industry on policy and research is less significant for public health than that of tobacco because (select all those that apply):	N of Responses	% of Respondents
Food products are less harmful than tobacco products at the individual level	64	68.8%
Food products are less harmful than tobacco products at the population level	54	58.1%
Food products are more socially acceptable in the contexts in which I work	53	57.0%
Food products are less socially acceptable in the contexts in which I work	4	4.3%
I personally find food products more acceptable/appealing	36	38.7%
There is less evidence that the food industry has attempted to influence policy in ways which are damaging to health	34	36.6%
There is less evidence that the food industry has attempted to influence research in ways which are damaging to health	28	30.1%
There is less evidence that the food industry has sought to mislead the public / policymakers about the negative health and social consequences of its products.	28	30.1%
Number of respondents		93

6. PERSONAL EXPERIENCES AND VIEWS		
1. Institutional affiliation	N of Responses	% of Respondents
University	103	38.7%
Knowledge-broker	18	6.8%
Charity	43	16.2%
Advocacy/campaigning	74	27.8%
Think tank	9	3.4%
Health service	48	18.0%
National government	21	7.9%
Local Government	12	4.5%
Other (Specify)	37	13.9%
Number of respondents		266

2. Which of these would you describe as a focus of your public health work? (please tick all that apply):	N of Responses	% of Respondents
Alcohol	89	33.0%
Food / obesity	105	38.9%
Global health	72	26.7%
Health inequalities	83	30.7%
Health systems	46	17.0%
Health policy	108	40.0%

Non-communicable diseases	91	33.7%
Tobacco control	109	40.4%
Other (Specify)	34	12.6%
Number of respondents		270

3. In what country do you primarily work?		
Country	N of Respondents	% of Respondents
UK	78	24.2
Canada	62	19.3
Australia	19	5.9
USA	11	3.4
India	9	2.8
Global	7	2.2
Brazil	6	1.9
New Zealand	5	1.6
Europe/EU	5	1.6
Other	60	18.6
N of respondents		262

4. Are you engaged in research?	N of Respondents	% of Respondents
Yes (Go to question 6)	151	55.9%
No (Skip question 6, go to question 7)	119	44.1%
Number of respondents	270	100.0%

5. If you are engaged in research, what methodological approach do you primarily employ?	N of Responses	% of Respondents
Qualitative methods	49	32.0%
Mixed methods	81	52.9%
Quantitative, non-experimental methods	55	35.9%
Quantitative, experimental methods	13	8.5%
Other (Specify)	3	2.0%
Number of respondents		153

6. In political matters, people talk of "the left" and "the right." How would you place your views on this scale, generally speaking?	N of Respondents	% of Respondents

‘1 Left’	19	7.1%
‘2’	55	20.6%
‘3’	68	25.5%
‘4’	28	10.5%
‘5’	25	9.4%
‘6’	10	3.7%
‘7’	10	3.7%
‘8’	5	1.9%
‘9’	0	0.0%
‘10 Right’	0	0.0%
Prefer not to say	47	17.6%
Number of respondents	267	100.0%

7. Are you affiliated with any of the following organisations/coalitions (tick all that apply)?	N of Responses	% of Respondents
Conflict of Interest Coalition	34	26.8%
Framework Convention Alliance	45	35.4%
Global Alcohol Policy Alliance (GAPA)	23	18.1%
NCD Alliance	44	34.6%
UK Centre for Tobacco and Alcohol Studies (UKCTAS)	16	12.6%
World Health Organization	54	42.5%
Number of respondents		127

8. Have you ever worked in any of the following industries? (tick all that apply)	N of Responses	% of Respondents
Alcohol	5	16.7%
E-cigarettes	0	0.0%
Food and beverage	19	63.3%
Pharmaceutical	13	43.3%
Tobacco	2	6.7%
Number of respondents		30

9. Do you have any formal relationship with, or financial investments in, any of the following industries? (tick all that apply)	N of Responses	% of Respondents
Alcohol	2	14.3%
E-cigarettes	0	0.0%
Food and beverage	8	57.1%
Pharmaceutical	5	35.7%
Tobacco	0	0.0%
Number of respondents		14

10. In the past ten years, have you received funding from any of the following sources for any research or other work relating to public health that you have undertaken / been professionally involved with (tick all that apply)?	N of Responses	% of Respondents
Alcohol industry	1	0.5%
E-cigarette industry	0	0.0%
Food and non-alcoholic beverage industry	10	5.1%
Publicly funded health services	68	34.3%
Local Government	50	25.3%
Health/medical charity/campaigning organisation	75	37.9%
Other charity/campaigning organisation	33	16.7%
Pharmaceutical industry	28	14.1%
Charitable foundation (such as Wellcome Trust, Bill & Melinda Gates Foundation	69	34.8%
Retail industry	5	2.5%
Tobacco industry	0	0.0%
National government	106	53.5%
Government funded research councils	67	33.8%
European Union	45	22.7%
International organisations	67	33.8%
Prefer not to say	2	1.0%
Other (Specify)	15	7.6%
Number of respondents		198

7. FINAL QUESTIONS		
1. Have you found it particularly difficult to answer our questions in relation to any of these industries? (please tick all that apply).	N of Responses	% of Respondents
NO	119	46.1%
YES, FOR ALCOHOL	36	14.0%
YES, FOR FOOD	94	36.4%
YES, FOR TOBACCO	19	7.4%
Number of respondents		258
